# Traditional Chinese Medicine Is Associated with Reduced Risk of Readmission in Rheumatoid Arthritis Patients with Anemia: A Retrospective Cohort Study

**DOI:** 10.1155/2022/4553985

**Published:** 2022-08-03

**Authors:** Yanqiu Sun, Jian Liu, Ling Xin, Jianting Wen, Qin Zhou, Xiaolu Chen, Xiang Ding, Xianheng Zhang

**Affiliations:** ^1^First Affiliated Hospital of Anhui University of Traditional Chinese Medicine, Hefei, Anhui 230038, China; ^2^Department of Rheumatology, First Affiliated Hospital of Anhui University of Traditional Chinese Medicine, Hefei 230038, Anhui Province, China; ^3^Institute of Rheumatology, Anhui Academy of Chinese Medicine, Hefei 230012, Anhui Province, China

## Abstract

**Objective:**

This study aimed to analyze the effect of traditional Chinese medicine (TCM) on the risk of readmission for rheumatoid arthritis (RA) patients with anemia.

**Methods:**

In this study, 893 hospitalized RA patients were followed up by telephone. A retrospective cohort study was conducted using propensity score matching (PSM). The Cox proportional hazards model was used to assess the influence of various factors on the risk of readmission for RA patients with anemia. The Kaplan–Meier survival curve was utilized to analyze the effect of TCM intervention time on readmission.

**Results:**

The incidence of anemia was 58.08% (471/811) in RA patients. After 1 : 1 PSM, 328 RA patients with anemia and 328 RA patients without anemia were finally included in our study. The readmission rate of anemia patients was higher than that of patients without anemia (*P* < 0.01). The readmission rate of RA patients with anemia was obviously lower in the TCM group than in the non-TCM group (*P* < 0.01). The Cox proportional hazards model showed TCM as an independent protective factor as it decreased the risk of readmission by 50% (HR = 0.50, 95% CI = 0.27–0.94, *P*=0.03) in RA patients with anemia. In addition, the risk of readmission was dramatically diminished in the high-exposure subgroup (TCM > 12 months) compared with the low-exposure subgroup (TCM ≤ 12 months) (log-rank *P*=0.016).

**Conclusion:**

TCM, as a protective factor, is associated with a reduced risk of readmission in RA patients with anemia.

## 1. Introduction

Rheumatoid arthritis (RA) is an autoimmune disease involving numerous systems, with symmetric joint pain and swelling as the main clinical manifestations, which can lead to joint deformity and loss of function [1]. RA is accompanied by multifaceted extra-articular manifestations, including rheumatoid nodules, vasculitis, pleural and pulmonary lesions, hematologic changes, cardiac lesions, renal lesions, neurological lesions, ocular lesions, and Sjogren's syndrome [2, 3]. Involvement of the blood system is common in multisystem damage caused by RA. Anemia is one of the most frequent extra-articular manifestations of RA with an incidence rate of approximately 30%–70% [4, 5]. In fact, anemia is considered a predictor of radiographic progression of RA and an indicator of inflammatory status during active disease [6, 7]. In addition, anemia can lead to a range of adverse manifestations, such as fatigue, weakened muscle strength, decreased physical activity, augmented hospitalizations, and reduced quality of life [8–10]. Therefore, anemia is an important factor affecting local joint injury and systemic symptoms in RA patients.

Currently, there is no radical cure for RA. The main drugs for the treatment of RA comprise immunosuppressants, nonsteroidal anti-inflammatory drugs (NSAIDs), and glucocorticoids, which, however, often contribute to adverse reactions, such as liver and kidney dysfunction and gastrointestinal injury [11]. In addition to anemia, interstitial lung disease and Sjogren's syndrome are also prevalent secondary lesions of RA [12]. Studies have shown that interstitial lung disease is associated with poor prognosis of RA and is considered a critical factor in the occurrence of death in RA [13, 14]. 8.7–30% of RA patients can be complicated with Sjogren's syndrome, which is an essential factor affecting the disease activity, treatment, and prognosis of RA [15–17]. Therefore, it is urgent to develop drugs for the treatment of RA and secondary lesions.

In recent years, traditional Chinese medicine (TCM) has achieved some accomplishments in treating RA by alleviating clinical symptoms and improving prognosis in RA [18, 19]. The application of meta-analysis, network pharmacology, and systems biology has tremendously facilitated research on TCM-related therapeutics, pharmacy, and molecules [20–22]. Therefore, we performed a retrospective and controlled cohort study combined with data mining technology to ascertain the impact of TCM on the incidence of adverse events in RA patients with anemia, thus providing a reference for the application of TCM in the treatment of RA.

## 2. Materials and Methods

### 2.1. Data Sources and Study Subjects

In this telephone-based follow-up cohort analysis, we reviewed the clinical data of 893 RA patients admitted to the Department of Rheumatology in the First Affiliated Hospital of Anhui University of Traditional Chinese Medicine from June 2013 to June 2021. These patients met the 2010 American College of Rheumatology/European Alliance of Associations for Rheumatology criteria for RA. The study was conducted in compliance with the principles of the Declaration of Helsinki. Our follow-up process fully protects the privacy of patients and did not interfere with the treatment option. The Ethics Committee of the First Affiliated Hospital of Anhui University of Traditional Chinese Medicine exempted patients from the right of informed consent (Review no. 2022MCZQ01).

**Figure 1:**
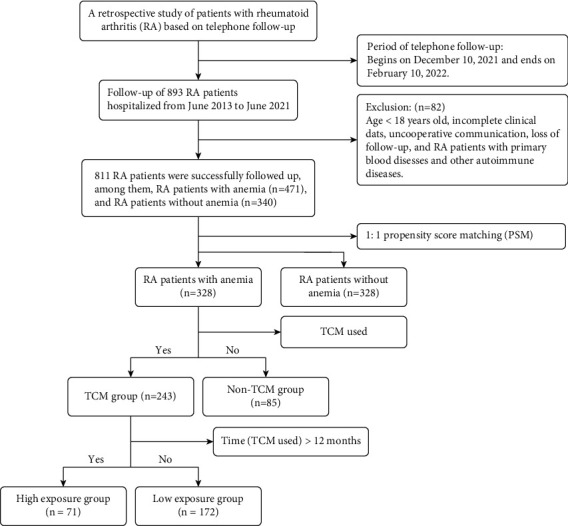
Flowchart of the study population.

A flowchart describes the selection process for patients with RA (Figure 1). The follow-up started on December 10, 2021, and ended on February 10, 2022. Subsequent to follow-up and screening, 811 RA patients were followed up successfully, among which 471 patients suffered from anemia, accounting for 58.08%. In order to balance the bias caused by the baseline data, RA patients were arranged into an anemia group (*n* = 328) and an RA without anemia group (*n* = 328) after propensity score matching (PSM). Subsequently, RA patients with anemia were clarified into a TCM group (*n* = 243) and a non-TCM group (*n* = 85) according to whether TCM was used or not. Finally, patients in the TCM group were assigned into a high-exposure subgroup (>12 months) and a low-exposure subgroup (≤12 months) based on the time of TCM intervention.

### 2.2. Contents of Telephone Follow-Up

Basic data of patients, such as name, age, gender, telephone number, and diagnostic information, were obtained through the hospital information system (HIS) and verified through phone tracking. The follow-up included basic medication and time, TCM use and time, basic diseases, and end point events.

Basic medication consisted of disease-modifying antirheumatic drugs (DMARDs; including methotrexate, leflunomide, and hydroxychloroquine), NSAIDs (including celecoxib, meloxicam, and lornoxicam), glucocorticoids (including methylprednisolone and prednisone acetate), and folic acid. Combinations of basic diseases were listed as follows: hypertension, diabetes, and hyperlipidemia. The main end points were as follows: the exacerbation of RA leads to readmission, interstitial lung disease caused by RA, Sjogren's syndrome secondary to RA, surgical treatment of joints, and death event. All follow-up personnel were professional doctors in the Department of Rheumatology. Each follow-up was inquired by one doctor and supervised and verified by two doctors at the same time.

### 2.3. Anemia Evaluation and Laboratory Examinations

According to the criteria of anemia defined by the World Health Organization, hemoglobin (Hb) < 130 g/L for men and <120 g/L for women. It is divided into 4 grades according to the degree of anemia: mild anemia is Hb < 130 g/L (male) or Hb < 120 g/L (female) and Hb > 90 g/L; moderate anemia is 60 g/L ≤ Hb ≤ 90 g/L; severe anemia is 30 g/L ≤ Hb < 60 g/L; and extremely severe anemia is Hb < 30 g/L. Laboratory examination indicators were detected for the enrolled patients: red blood cell (RBC) count, Hb, serum Ferrum (Fe), erythrocyte sedimentation rate (ESR), C-reactive protein (CRP), rheumatoid factor (RF), *α*1-acid glycoprotein (*α*1-AGP), anticyclic citrullinated peptide (anti-CCP) antibody, immunoglobulin A (IgA), immunoglobulin G (IgG), and immunoglobulin M (IgM), complement component 3 (C3), complement component 4 (C4), serum creatinine (CREA), blood urea nitrogen (BUN), uric acid (UA), alanine aminotransferase (ALT), and aspartate transferase (AST).

### 2.4. Cox Proportional Hazards Model Analysis

In this study, the readmission of RA patients with anemia was used as the dependent variable, while age, gender, combined diseases, basic drugs, and TCM were utilized as independent variables. Combined with the time of readmission and follow-up, the Cox proportional hazards model analysis was carried out. A univariate analysis was performed to obtain preliminary results. A further multivariate analysis was conducted to study the independent influencing factors for readmission.

### 2.5. Association Rule Analysis

The specific TCM was defined as T and the unused TCM as F. After TCM treatment, the decrease of ESR, CRP, RF, anti-CCP, IgA, IgG, IgM, C3, and C4 was recorded as T, whereas their stability and increase was F. Hb, RBC, and Fe index values were regarded as T when they increased and F when they remained unchanged or decreased. Patient readmission was defined as F and no readmission was defined as T. This assisted in discovering the correlation between TCM and the observed indicator or with readmission. The specific calculation formula of association rule analysis refers to the research of Huang et al. [23].

### 2.6. Statistical Analysis

Count data as numbers or percentages and chi-squared test was used for comparison between groups. Continuous data were represented by median [quartile difference (IQR)] and comparisons were made using the rank-sum test. SPSS 24.0 was used for data statistics, Cox proportional risk model analysis, and Kaplan–Meier (K-M) survival curve analysis. There was a statistically significant difference when *P* < 0.05.

## 3. Results

### 3.1. Demographic Characteristics of RA Patients with or without Anemia

A total of 811 RA patients were included in this study, including 471 (58.08%) patients with anemia and 340 (41.92%) patients without anemia. There were significant differences in hypertension, hyperlipidemia, and folic acid use between the two groups (*P* < 0.05).

In order to avoid the bias caused by baseline, the method of 1 : 1 PSM was utilized to balance the bias between the two groups in terms of combined diseases and basic medication. Subsequent to matching, 656 RA patients were included, including 328 RA patients with anemia and 328 RA patients without anemia. No obvious difference was observed between the two groups regarding age, gender, combined diseases, and basic medication (*P* > 0.05). In terms of end point events, the incidence of readmission, interstitial lung disease, and surgical treatment was noticeably higher in RA patients with anemia than in RA patients without anemia (*P* < 0.05). In addition, Sjogren's syndrome and death were not statistically different between the two groups (*P* > 0.05; Table 1).

### 3.2. Demographic Characteristics of RA Patients with Anemia in the TCM Group and the Non-TCM Group

The 328 patients with RA anemia were allocated into the TCM group (*n* = 243) and the non-TCM group (*n* = 85). There existed no considerable difference in gender, age, combined diseases, anemia classification, and the incidence of interstitial lung disease, Sjogren's syndrome, surgical treatment, and death between the two groups (*P* > 0.05). In the TCM group, TCM was used in 80 cases for 0–6 months, 92 cases for 6–12 months, and 71 cases for more than 12 months. Compared with the non-TCM group, the incidence of readmission in the TCM group was evidently decreased (*P* < 0.01; Table 2).

### 3.3. Influencing Factors for the Readmission of RA Patients with Anemia

The Cox proportional risk model was used to determine the risk factors for readmission in RA patients with anemia (Table 3). The univariate analysis manifested that the TCM group had a substantially lower readmission than in the TCM group [Hazards Ratio (HR) = 0.50, 95% CI = 0.30–0.84, *P*=0.01]. Patients administering DMARDs had a prominently lower readmission risk (HR = 0.35, 95% CI = 0.16–0.77, *P*=0.01). Likewise, the readmission risk also was lower in patients receiving NSAID treatment (HR = 0.59, 95% CI = 0.35–0.99, *P*=0.05). Furthermore, the multivariate analysis was implemented to screen the independent factors influencing the readmission of RA patients with anemia. The results exhibited that the risk of readmission in patients of the TCM group was reduced by 50% (HR = 0.50, 95% CI = 0.27–0.94, *P*=0.03) compared with the patients of the non-TCM group. These results illustrated that the use of TCM was a protective factor to diminish the readmission rate of RA patients with anemia.

### 3.4. The K-M Survival Curve Analysis of the Effects of TCM on the Readmission of RA Patients with Anemia

**Figure 2:**
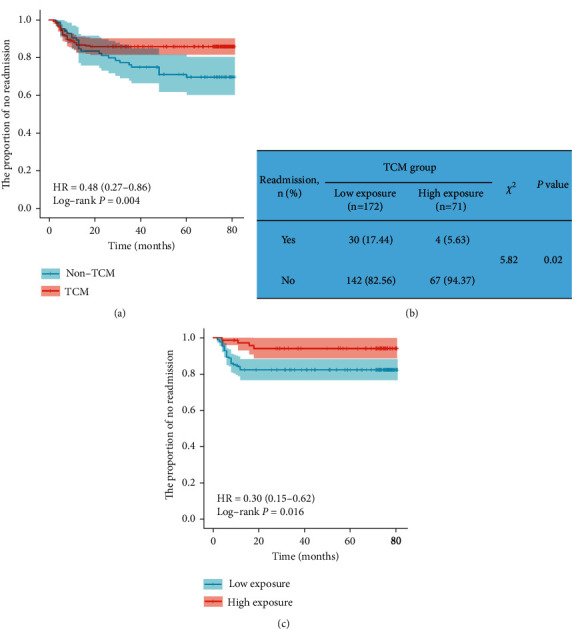
The K-M survival curve of readmission in RA patients with anemia. (a) The K-M survival curve was used to analyze the effect of TCM on the risk of readmission. (b) Incidence of readmission in low-exposure group and high-exposure group. (c) The K-M survival curve was utilized to assess the effect of TCM intervention time on the risk of readmission.

The K-M survival curve was adopted to compare the risk of readmission between the TCM group and the non-TCM group and further delve into the impact of TCM intervention time on the risk of readmission. Specifically, the risk of readmission in the TCM group was remarkably lower than that in the non-TCM group (log-rank *P*=0.004; Figure 2(a)). In the TCM group, the TCM intervention time of ≤12 months was defined as low exposure and the TCM intervention time of >12 months as high exposure. The readmission rate was significantly lower in the high-exposure group compared with the low-exposure group (*P*=0.02; Figure 2(b)). In addition, in contrast to the low-exposure group, the high-exposure subgroup had a significantly lower risk of readmission (log-rank *P*=0.016; Figure 2(c)).

### 3.5. Characteristics of TCM in RA Patients with Anemia

The top 20 TCMs used by RA patients with anemia can be classified into five categories, including spleen-strengthening and dampness-removing drugs, heat-clearing and dampness-removing drugs, wind-dispelling and dehumidification drugs, blood-activating and stasis-removing drugs, and deficiency-tonifying drugs. The top 2 used Chinese patent medicines were Xinfeng capsules (XFC) and Huangqin Qingre Chubi capsules (HQC) (Supplementary Table S1).

### 3.6. Effects of TCM on Immune Inflammatory Indexes and Erythrocyte Parameters in RA Patients with Anemia

In the non-TCM group, compared with before treatment, Hb and RBC levels increased (*P* < 0.01), ESR, CRP, *α*1-AGP, IgG, C3, and C4 levels decreased (*P* < 0.01) after treatment. In TCM group, compared with before treatment, RBC and Hb levels were elevated (*P* < 0.01), and ESR, CRP, RF, *α*1-AGP, anti-CCP, IgA, IgG, C3, and C4 levels were reduced (*P* < 0.05) after treatment. In addition, TCM group was significantly better than non-TCM group in reducing RF level (*P* < 0.05; Table 4).

### 3.7. Effects of TCM on Kidney and Liver Function in RA Patients with Anemia

In non-TCM group, compared with before treatment, the level of UA decreased significantly after treatment (*P* < 0.01; Table 5). In TCM group, compared with before treatment, the level of UA decreased significantly after treatment (*P* < 0.01), and there was no statistical significance in the levels of CREA, BUN, ALT, and AST (*P* > 0.05; Table 5). There was no significant difference in the level of reduced UA between the two groups (*P* > 0.05; Table 5).

### 3.8. Association Rules Analysis of the Correlation between TCM Treatment and Laboratory Indicators in RA Patients with Anemia

The association rule analysis was carried out with the TCM used as the antecedent and the improved laboratory indicators as the consequent. The results showed that Salvia, Poria, Angelicae Sinensis, Cyathulae radix, XFC, HQC, and other drugs were strongly correlated with the improvement of ESR, CRP, RF, *α*1-AGP, anti-CCP, RBC, and Hb. In the above results, the support is greater than 20%, the confidence is greater than 60%, and the lift is greater than 1 (Table 6).

### 3.9. Association Rule Analysis between TCM and Readmission or Not in RA Patients with Anemia

To investigate whether TCM is associated with readmission in RA patients with anemia, we defined readmission as F and no readmission as T. The results of association rule analysis showed that Spatholobi Caulis, XFC, Pinellia Ternata, Lonice Raejaponicae Caulis, Curcumae Radix, and Spatholobi Caulis were strongly associated with no readmission. The associated support is greater than 30%, the confidence is greater than 85%, and the lift is greater than 1 (Table 7).

## 4. Discussion

Anemia induced by RA is majorly manifested as anemia of chronic disease (ACD) or inflammatory anemia, and the degree of anemia is mostly mild and moderate [24]. ACD in RA is attributed to a number of pathogenic mechanisms, such as the increased release of inflammatory cytokines, the disorder of iron metabolism, and the obstacle of erythropoiesis [25]. Our previous studies elucidated that the high-level immune inflammatory response in women was a risk factor for the increase of sharp scores in RA patients with anemia [26]. Similarly, increasing studies evidenced that inflammatory anemia might be an indicator of disease activity and structural damage in RA patients [7] and that RA patients with anemia have a longer hospital stay, more surgery times, and higher hospitalization costs [27]. Our study suggested that the incidence of anemia was 58.08% (471/811), consistent with the previous relevant literature [28]. The incidence of readmission was significantly higher in RA patients with anemia than in RA patients without anemia. Furthermore, TCM treatment contributed to declines in the risk of readmission in RA patients with anemia, and the longer the TCM use, the lower the risk of readmission.

TCM recently has attracted elevating attention from clinicians and relevant researchers for its role in the treatment of RA [29]. Former research has uncovered that the risk of fracture has been reduced by more than 50% in RA patients undergoing over 2 years of TCM treatment [30]. Chiu et al. noted that compared with the non-TCM group, the prognosis of the circulatory system of inpatients was favorable in the auxiliary TCM group, accompanied by two-thirds of reductions in the readmission rate of circulatory system events [31]. Our data unveiled that TCM effectively augmented the levels of RBC and Hb and reduced the levels of ESR, hs-CRP, RF, *α*1-AGP, anti-CCP, IgA, IgG, and UA in RA patients with anemia. In addition, it is worth noting that TCM intervention has no significant effect on liver and kidney function. The Cox proportional hazards regression analysis elaborated TCM intervention as the protective factor for readmission. The subsequent K-M survival curve analysis disclosed that not only did TCM reduce the risk of readmission of RA patients with anemia, but also the longer the use of TCM (>12 months), the lower the risk of readmission. In China, the combination of TCM and antirheumatism has been affirmed in the evaluation of clinical efficacy and safety [32], especially in repressing gastrointestinal diseases, abnormal liver function, leucopenia, skin allergy and rash, headache and dizziness, hair loss, and other adverse reactions.

The combined use of TCM is a common way to treat diseases, which often exerts the effect of increasing efficiency and reducing toxicity [33]. Multicomponents, multitargets, and multichannels are the characteristics of TCM in the treatment of RA and anemia [34]. The present study unraveled that the top 20 TCMs used by RA patients with anemia could be categorized into spleen-strengthening and dampness-removing drugs, heat-clearing and dampness-removing drugs, wind-dispelling and dehumidification drugs, blood-activating and stasis-removing drugs, and deficiency-tonifying drugs. Wang et al. [35] analyzed 302 RA patients related to “Zheng” in TCM, which indicated that the specific Zheng was associated with the characteristics of RA and that eliminating dampness, cooling patients, and promoting blood circulation might assist in treating severe RA. The association rules analysis in the present research manifested that Salvia, Poria, Angelicae Sinensis, Cyathulae radix, and Chinese patent medicine XFC and HQC were tightly correlated with the improvement of immune inflammatory indicators and erythrocyte parameters. In addition, further analysis showed that Spatholobi Caulis, XFC, Pinellia Ternata, Lonice Raejaponicae Caulis, Curcumae Radix, and Spatholobi Caulis were strongly associated with no readmission. Modern pharmacological research suggests that Poria has an excellent regulatory function in anti-inflammation and immune function [36]. Salvia miltiorrhiza exhibits definite curative effects in facilitating anti-inflammation, improving circulation, and reducing cardiovascular events [37]. Chen et al. [38] have observed that TCM has an obvious curative effect on improving anemia, among which Astragalus membranaceus and cinnamon decoction are the most frequently utilized single herbs and prescriptions in TCM for the treatment of anemia. The randomized controlled trial of XFC and leflunomide in the treatment of RA elucidated that XFC not only had superior efficacy and safety but also could improve the patient perception score [39]. XFC has been validated to be effective in improving extra-articular manifestations, such as cardiopulmonary function, anemia, and lipid metabolism disorders [40, 41]. In addition, HQC also assumes an established role in diminishing immune inflammatory responses and suppressing oxidative damage in RA [42].

Several limitations are present in this study. Only a single research institution is involved, and female patients account for more than 80% of the research population. Therefore, the research results may not be fully applicable to male RA patients. TCM formula is composed of many components, the specific active components are not clear, and the quality control is faced with some challenges. In addition, the combination of various herbs may cause gastrointestinal reactions in patients, increase the metabolic burden of liver and kidney, and potential adverse reactions.

In conclusion, TCM as a protective factor is associated with a reduced risk of readmission in RA patients with anemia. Furthermore, the longer the TCM use, the lower the risk of readmission. Although it is difficult to define whether this result is only caused by TCM, this study provides a reference for more comprehensive and extensive research in the future.

## Figures and Tables

**Table 1:** Comparison of demographic characteristics between RA patients with and without anemia.

Characteristic	Before PSM matched			*P* value	After PSM matched			*P* value
	Total (*n* = 811)	RA with anemia (*n* = 471)	RA without anemia (*n* = 340)		Total (*n* = 656)	RA with anemia (*n* = 328)	RA without anemia (*n* = 328)	
Age (years), *n* (%)								
20–60	470 (57.95)	261 (55.41)	209 (61.47)	0.09	384 (58.54)	185 (56.40)	199 (60.67)	0.27
>60	341 (42.05)	210 (44.59)	131 (38.53)		272 (41.46)	143 (43.60)	129 (39.33)	
Median (IQR)	56 (49, 66)	56 (49, 67)	56 (49, 65)	0.29	56 (49, 66)	56 (48, 67)	56 (49, 65)	0.79

Gender, *n* (%)								
Female	673 (82.98)	392 (83.23)	281 (82.65)	0.83	539 (82.16)	270 (82.32)	269 (82.01)	0.92
Male	138 (17.02)	79 (16.77)	59 (17.35)		117 (17.84)	58 (17.68)	59 (17.99)	

Combined diseases, *n* (%)								
Hypertension	176 (21.70)	116 (24.63)	60 (17.65)	0.02	129 (19.66)	69 (21.04)	60 (18.29)	0.38
Diabetes	34 (4.19)	21 (4.46)	13 (3.82)	0.66	25 (3.81)	13 (3.96)	12 (3.66)	0.84
Hyperlipidemia	91 (11.22)	41 (8.70)	50 (14.71)	0.01	76 (11.59)	37 (11.28)	39 (11.89)	0.81

Basic medicine, *n* (%)								
DMARDs	767 (94.57)	448 (95.12)	319 (93.82)	0.42	617 (94.05)	309 (94.21)	308 (93.90)	0.87
NSAIDs	424 (52.28)	253 (53.72)	171 (50.29)	0.34	328 (50.00)	158 (48.17)	170 (51.83)	0.35
Glucocorticoids	202 (24.91)	118 (25.05)	84 (24.71)	0.91	174 (26.52)	90 (27.44)	84 (25.61)	0.60
Folic acid	241 (29.72)	160 (33.97)	81 (23.82)	<0.01	148 (22.56)	67 (20.43)	81 (24.70)	0.19

End point events, *n* (%)								
Readmission	107 (13.19)	75 (15.92)	32 (9.41)	0.01	87 (13.26)	59 (17.99)	28 (8.54)	<0.01
Interstitial lung disease	94 (11.59)	66 (14.01)	28 (8.24)	0.01	78 (11.89)	50 (15.24)	28 (8.54)	0.01
Sjogren's syndrome	80 (10.11)	55 (11.68)	25 (7.35)	0.04	56 (8.54)	32 (9.76)	24 (7.32)	0.26
Surgical treatment	60 (7.40)	46 (9.77)	14 (4.12)	<0.01	45 (6.86)	31 (9.45)	14 (4.27)	0.01
Death	21 (2.59)	15 (3.18)	6 (1.76)	0.21	15 (2.29)	9 (2.74)	6 (1.83)	0.43

*Note.* PSM: propensity score matching; DMARDs: disease-modifying antirheumatic drugs; NSAIDs: nonsteroidal anti-inflammatory drugs.

**Table 2:** Comparison of demographic characteristics of RA patients with anemia between the TCM group and the non-TCM group.

Characteristic	TCM group (*n* = 243)	Non-TCM group (*n* = 85)	*P* value
Age (years), *n* (%)			
20–60	130 (53.50)	55 (64.71)	0.07
>60	113 (46.50)	30 (35.29)	

Gender, *n* (%)			
Female	200 (82.30)	70 (82.35)	0.99
Male	43 (17.70)	15 (17.65)	

TCM (months), *n* (%)			
0	0 (0)	85 (100)	—
>0, ≤6	80 (32.92)	NA	
>6, ≤12	92 (37.86)	NA	
>12	71 (29.22)	NA	

Anemia classification, *n* (%)			
Mild anemia	201 (82.72)	69 (81.18)	0.75
Moderate anemia	38 (15.64)	16 (18.82)	0.50
Severe anemia	4 (1.65)	0 (0)	—

Combined diseases, *n* (%)			
Hypertension	54 (22.23)	15 (17.65)	0.37
Hyperlipidemia	30 (12.35)	7 (8.24)	0.30
Diabetes	8 (3.29)	5 (5.88)	0.29

End event, *n* (%)			
Readmission	34 (13.99)	25 (29.41)	<0.01
Interstitial lung disease	39 (16.05)	11 (12.94)	0.42
Sjogren's syndrome	20 (8.23)	12 (14.12)	0.12
Surgical treatment	22 (9.05)	9 (10.59)	0.68
Death	7 (2.88)	2 (2.35)	1.00

*Note.* TCM: traditional Chinese medicine.

**Table 3:** The Cox proportional hazards model for the readmission of RA patients with anemia.

Variables	Univariate			Multivariate		
	HR	95% CI	*P* value	HR	95% CI	*P* value
Age (year)						
20–60	1			1		
≥60	1.13	0.64–1.89	0.65	1.26	0.69–2.27	0.45

Gender						
Male	1			1		
Female	1.05	0.53–2.07	0.90	1.03	0.52–2.06	0.93

TCM used						
No	1			1		
Yes	0.50	0.30–0.84	0.01	0.50	0.27–0.94	0.03
Hypertension	1.23	0.68–2.22	0.50	1.42	0.72–2.78	0.31
Diabetes	0.72	1.18–2.98	0.65	0.52	0.11–2.33	0.39
Hyperlipidemia	1.07	0.46–2.50	0.88	1.00	0.41–2.39	0.09
DMARDs	0.35	0.16–0.77	0.01	0.48	0.19–1.25	0.13
NSAIDs	0.59	0.35–0.99	0.05	0.58	0.32–1.05	0.07
Glucocorticoids	0.96	0.54–1.70	0.88	0.53	0.27–1.04	0.07
Folic acid	1.39	0.75–2.57	0.30	1.69	0.88–3.25	0.11

*Note.* HR: hazards ratio; 95% CI: 95% confidence interval; TCM: traditional Chinese medicine; DMARDs: disease-modifying antirheumatic drugs; NSAIDs: nonsteroidal anti-inflammatory drugs.

**Table 4:** Effects of TCM on immune inflammatory indexes and erythrocyte parameters in RA patients with anemia.

Index	Non-TCM group (*n* = 85)		TCM group (*n* = 243)	
	Before treatment	After treatment	Before treatment	After treatment
Hb (g/L)	99.00 (93.50, 108.00)	103.00 (95.00, 110.50)^*∗∗*^	105.00 (95.00, 111.00)	108.00 (99.00, 115.00)^##^
RBC (10^12^/L)	3.67 (3.41, 4.00)	3.87 (3.67, 4.23)^*∗∗*^	3.72 (3.45, 4.04)	3.85 (3.58, 4.22)^##^
Fe (*μ*mol/L)	7.79 (5.47, 11.49)	8.50 (6.05, 12.28)	9.70 (6.31, 13.05)	9.96 (6.70, 13.30)
ESR (mm/h)	41.00 (26.00, 57.50)	28.00 (18.50, 42.50)^*∗∗*^	41.00 (21.00, 68.00)	24.00 (14.00, 44.00)^##^
CRP (mg/L)	14.79 (3.77, 47.14)	2.23 (0.58, 8.93)^*∗∗*^	9.99 (1.90, 36.84)	1.45 (0.48, 225.50)^##^
RF (U/mL)	92.20 (34.55, 235.50)	87.90 (32.65, 238.25)	113.10 (32.90, 238.30)	98.60 (29.00, 201.80)^##△^
*α*1-AGP (mg/dL)	127.00 (96.00, 153.50)	106.00 (84.00, 137.50)^*∗∗*^	118.00 (91.00, 154.00)	101.00 (81.00, 130.00)^##^
Anti-CCP (U/mL)	235.92 (27.75, 559.00)	134.09 (27.23, 551.64)	245.00 (64.17, 525.27)	220.38 (49.17, 500.41)^#^
IgA (g/L)	2.58 (1.78, 3.12)	2.34 (1.77, 3.12)	2.29 (1.65, 3.18)	2.18 (1.64, 3.08)^##^
IgG (g/L)	12.40 (9.67, 15.05)	12.40 (9.72, 14.95)^*∗∗*^	12.30 (9.50, 15.49)	11.75 (9.20, 14.80)^##^
IgM (g/L)	1.27 (0.94, 1.68)	1.25 (0.92, 1.71)	1.20 (0.78, 1.63)	1.19 (0.81, 1.64)
C3 (g/L)	103.40 (83.20, 120.25)	99.40 (83.70, 114.85)^*∗∗*^	104.20 (87.70, 122.30)	100.80 (89.70, 115.10)^##^
C4 (g/L)	21.80 (16.85, 28.30)	20.90 (15.35, 25.15)^*∗∗*^	22.30 (17.20, 28.70)	21.50 (15.60, 25.90)^##^

*Note.* Compared with the non-TCM group before treatment, ^*∗∗*^*P* < 0.01. Compared with the TCM group before treatment, ^#^*P* < 0.05 and ^##^*P* < 0.01. Compared with the non-TCM group difference (after treatment − before treatment), ^Δ^*P* < 0.05. TCM: traditional Chinese medicine; Hb: hemoglobin; RBC: red blood cell count; Fe: serum Ferrum; ESR: erythrocyte sedimentation rate; CRP: C-reactive protein; RF: rheumatoid factor; *α*1-AGP: *α*1-acid glycoprotein; anti-CCP: anticyclic citrullinate peptide antibody; IgA: immunoglobulin A; IgG: immunoglobulin G; IgM: immunoglobulin M; C3: complement component 3; C4: complement component 4.

**Table 5:** Effects of TCM on kidney and liver function in RA patients with anemia.

Index	Non-TCM group (*n* = 85)		TCM group (*n* = 243)	
	Before treatment	After treatment	Before treatment	After treatment
CREA (*μ*mol/L)	46.50 (39.10, 51.50)	43.80 (39.65, 55.50)	49.20 (43.90, 57.30)	49.80 (43.10, 55.80)
BUN (mm/h)	4.23 (3.29, 5.18)	4.58 (3.61, 5.63)	4.91 (3.62, 5.80)	4.99 (3.90, 6.19)
UA (mg/L)	211.00 (170.00, 263.50)	196.00 (145.00, 236.50)^*∗∗*^	254.00 (202.00, 306.00)	226.00 (187.00, 273.00)^##^
ALT (g/L)	11.00 (8.00, 15.50)	13.00 (10.00, 19.00)	12.00 (9.00, 18.00)	13.00 (9.00, 20.00)
AST (g/L)	15.00 (13.00, 19.50)	16.00 (13.00, 21.00)	16.00 (13.00, 21.00)	15.00 (13.00, 20.00)

*Note.* Compared with the non-TCM group before treatment, ^*∗∗*^*P* < 0.01. Compared with the TCM group before treatment, ^##^*P* < 0.01. CREA: serum creatinine; BUN: blood urea nitrogen; UA: uric acid; ALT: alanine aminotransferase; AST: aspartate transferase.

**Table 6:** Analysis of association rules between TCM treatment and laboratory indicators in RA patients with anemia.

Number	The aforesaid	The consequent	Support (%)	Confidence (%)	Lift
1	Salvia	ESR↓	83.54	63.55	1.02
2	Poria	ESR↓	85.60	65.38	1.05
3	Salvia and poria	ESR↓	75.31	66.67	1.07
4	HQC	ESR↓	24.28	62.71	1.01
5	Dandelion and salvia	CRP↓	64.20	73.72	1.01
6	XFC	CRP↓	31.28	73.68	1.01
7	Spatholobi caulis	RF↓	44.03	64.49	1.03
8	Spatholobi caulis and salvia	RF↓	39.09	64.21	1.03
9	XFC	RF↓	31.28	64.47	1.03
10	XFC	*α*1-AGP↓	31.28	60.53	1.14
11	XFC and salvia	*α*1-AGP↓	29.22	60.56	1.14
12	Hordei fructus germinatus	anti-CCP↓	24.28	61.02	1.24
13	Hordei fructus germinatus and poria	anti-CCP↓	23.87	60.34	1.22
14	Poria	RBC↑	85.60	60.10	1.03
15	Coicis semen and poria	RBC↑	70.78	61.05	1.04
16	Cyathulae radix	RBC↑	25.93	60.32	1.03
17	Angelicae sinensis	RBC↑	28.81	64.29	1.10
18	Salvia and poria	Hb↑	75.31	61.20	1.01
19	Persicae semen and salvia	Hb↑	65.02	62.03	1.03
20	Angelicae sinensis	Hb↑	28.81	64.29	1.06

*Note.* XFC: Xinfeng capsules; HQC: Huangqin Qingre Chubi capsules; ESR: erythrocyte sedimentation rate; CRP: C-reactive protein; RF: rheumatoid factor; *α*1-AGP: *α*1-acid glycoprotein; anti-CCP: anti-cyclic citrullinate peptide antibody; RBC: red blood cell count; Hb: hemoglobin.

**Table 7:** The category of TCM associated with no readmission in RA patients with anemia.

Number	The aforesaid	The consequent	Support (%)	Confidence (%)	Lift
1	Spatholobi caulis	No readmission	44.03	89.72	1.04
2	XFC	No readmission	31.28	88.16	1.02
3	Pinellia ternata	No readmission	43.21	87.62	1.02
4	Lonice raejaponicae caulis	No readmission	41.15	87.00	1.01
5	Curcumae radix	No readmission	34.16	86.75	1.01

*Note.* XFC: Xinfeng capsules.

## Data Availability

The data used in this study are available from the corresponding author upon reasonable request.

## Supplementary Materials

Supplementary Materials
*Supplementary Table S1*. TCM characteristics in treating rheumatoid arthritis patients with anemia.
