# Supplementary material for: Traditional Chinese Medicine Is Associated with Reduced Risk of Readmission in Rheumatoid Arthritis Patients with Anemia: A Retrospective Cohort Study
Source: Evid Based Complement Alternat Med. 2022 Aug 3;2022:4553985. doi: 10.1155/2022/4553985 (PMC9365533; doi:10.1155/2022/4553985)
Supplement: Supplementary Materials — Supplementary Table S1. TCM characteristics in treating rheumatoid arthritis patients with anemia. [file 4553985.f1.docx]

**Supplementary Table S1 TCM characteristics in treating rheumatoid arthritis patients with anemia**

| Category | Herb | Number | Nature and taste | Meridian tropism |
| --- | --- | --- | --- | --- |
| Drugs for invigorating spleen and removing dampness | Poria | 208 | Sweet, light, flat | Spleen, kidney |
|  | Citri reticulatae pericarpium | 200 | Bitter, hot, warm | Lung, spleen |
|  | Coicis semen | 191 | Sweet, light, cold | Spleen, stomach, lung |
|  | Pinellia ternata | 105 | Pungent, warm | Spleen, stomach, lung |
|  | Magnolia officinalis | 68 | Bitter, pungent, warm | Spleen, stomach, lung, arge intestine |
|  | Setariae fructus germinatus | 63 | Sweet, warm | Spleen, stomach |
|  | Hordei fructus germinatus | 59 | Swee，flat | Spleen, stomach |
| Antipyretic and diuretic drugs | Dandelion | 173 | Bitter, sweet, cold | Liver, stomach |
|  | Alisma orientalis | 124 | Sweet, light, cold | Kidney, bladder |
|  | Asiatic plantain | 69 | Sweet, cold | Liver, kidney, bladder |
| Drugs for dispelling wind and dampness | Lonice raejaponicae caulis | 100 | Sweet, cold | Lung, stomach |
|  | Radix Cynanchi Paniculati | 74 | Bitter, pungent, cold | Liver, kidney, bladder |
|  | Cyathulae radix | 63 | Sweet, bitter, flat | Liver, kidney |
| Drugs for promoting blood circulation and removing blood stasis | Salvia | 203 | Bitter, slightly cold | Heart, liver |
|  | Persicae semen | 164 | Bitter, sweet, flat | Heart, liver, large intestine |
|  | Curcumae radix | 83 | Pungent, bitter, cold | Liver, heart and lung |
| Tonic medicine | Spatholobi caulis | 107 | Bitter, sweet, warm | Liver, kidney |
|  | Angelicae sinensis radix | 70 | Sweet, pungent, warm | Liver, heart, spleen |
|  | Astragali radix | 69 | Sweet, mild | Lung, spleen |
| Other | Licorice | 188 | Sweet, flat | Lung, spleen, stomach |
| Chinese patent medicine | Xinfeng Capsule (XFC) | 76 | Replenish qi and spleen, remove dampness and dredge collaterals | |
|  | Huangqin Qingre Chubi capsule (HQC) | 59 | Clearing heat and dampness, dispelling wind and relieving pain | |
